# Review of domestic and international methods of measuring radon in residential buildings

**DOI:** 10.1186/s40557-016-0097-0

**Published:** 2016-03-22

**Authors:** Cheolmin Lee, Dajeong Lee

**Affiliations:** SeoKyeong University, 124 Seogyeong-ro, Seongbuk-gu, Seoul 02173 South Korea; Hanyang Universtiy, 306, 130 Gwangnaru-ro, Seongdong-Gu, Seoul 04788 South Korea

## Abstract

Radon, a source of natural background radiation, has been a subject of extensive studies as a major causative agent of lung cancer at the domestic and international levels. This study investigated and compared domestic and international methods of radon measurement. In the United States, radon is measured through primary and secondary testing, and a similar method is used in Canada. In the United Kingdom, only long-term radon measurements are taken with seasonal adjustments. In the Republic of Korea, both long-term and short-term measurements are taken with only primary testing. Through this study, standards for domestic radon measurement methods and improvement plans could be suggested.

## Background

Radon is a natural radioactive substance found in nature, and is known to be a major causative agent of lung cancer. Worldwide, the effect of radon on the development of lung cancer and related deaths have been evaluated and results have been reported. However, in the Republic of Korea, such scientific and systematic reports are significantly lacking except for few predictive reports of lung cancer mortality in the country published by the Atomic Energy Research Institutes and some researchers considering the national population and lung cancer mortality rate due to radon in foreign countries. Therefore, research in this field is an urgent requirement.

Currently in Korea, there are no radon concentration standards for houses except for public facilities. Considering this circumstance, examination of official methods for measuring radon in houses is necessary.

In order to examine measurement methods that can be used in houses and deduce improvement plans for the existing official method, this study investigated radon measurement protocols used in the United States, Canada, and the United Kingdom, and compared them to that used in Korea.

## Review

### Republic of Korea

The domestic radon measurement method and standards are specified in ‘Indoor Air Quality Control in Public Use Facilities, etc. Act’ for detecting radon in air in public facilities. Under this regulation, the recommended level of radon is 148 Bq/m^3^, which is the same as that in the US. The primary test method involves long-term measurement in public facilities for over a period of 90 days or longer using a passive detector, and the secondary test method involves short-term measurements for over periods of 2 to 10 days using a continuous detection device.

### The United States

The Unites States Environmental Protection Agency (EPA) published ‘Protocols for Radon and Radon Decay Product Measurements in Homes’ [1] by studying and summarizing ‘A Citizen's Guide to Radon: The Guide to Protecting Yourself and Your Family from Radon’ [2] and ‘Home Buyer's and Seller's Guide to Radon’ [3]. Through this report, Indoor Radon and Radon Decay Product Measurement Device Protocols [4], a technical report explaining the measurement technology was published and presented in 1992. Radon is measured through separate primary and secondary testing.

Primary testing is recommended as initial short-term measurements by homeowners. Short-term measurements can be taken easily and quickly to help save cost as well as draw a decision on radon reduction for health protection. The period of short-term measurement ranges from 48 h to 7 days depending on the manual.

These measurements must be taken in sealed buildings in order to stabilize radon and radon daughter concentration. Further, air exchange systems (conditioning systems or window fans) connected to the outside must be shut down in the entire house. For a measurement period of 4 days, the building must be sealed for at least 12 h before the start of the measurement. During late fall and early spring, when windows are closed and heating systems are not used, more stable radon levels are observed in some houses.

Rapid changes in atmospheric pressure alter the speed of radon inflow and generate drastic changes between indoor and outdoor pressure. Weather with abnormal seasonal changes or impacts due to other effects should be considered during the measurement.

Secondary testing might not be required for radon levels of less than 4 pCi/L. While this level is acceptable without further radon reduction, homeowners may choose to retest. New tests must be performed when living patterns of homeowners change, houses are remodeled, or basements are used as living spaces.

Even with less than 4 pCi/L of radon concentration, there are partial dangers. When the average radon level is 2–4 pCi/L in both primary and secondary short-term measurements or secondary long-term measurement, homeowners are recommended to consider radon reduction. Although the technology of reducing indoor radon level has not been established in all houses yet, it can be reduced to 2 pCi/L or less in certain houses. When the level is 4 pCi/L or higher in short-term measurements, residents must perform short-term or long-term secondary measurements.

The purpose of the secondary measurement is to provide sufficient information for homeowners to decide whether they need to perform radon reduction. Long-term or short-term secondary measurements confirm whether radon reduction is required and provide additional information to the primary testing. When long-term tracking observations are performed, the results must be representative of the average value of the long-term radon measurement. Secondary testing also provides confirmation of the primary test results.

Secondary testing, which can be performed with either short-term or long-term detection devices, must be performed at the same location as the primary testing. Long-term measurements (90 days or longer) allow more accurate measurements of annual average of radon level than short-term measurements. However, if radon level in the primary testing is high (10 pCi/L or higher) or quick results are required, the EPA recommends short-term testing. This will allow the homeowner to gain information required to make a prompt decision on the necessity of reduction. When radon level is 4–10 pCi/L in the primary testing, either short-term or long-term measurements can be taken.

When the radon level is 4 pCi/L or higher in the secondary testing, the EPA recommends improvement measures. In this manner, when it remains the same or at 4 pCi/L or higher, radon reduction is recommended. Although secondary short-term measurements must be performed in a sealed building much like in primary testing, this condition is not necessary for long-term (90 days or longer) measurement.

### Canada

While the Canadian government does not have regulations on the acceptable level of radon, Health Canada developed a guideline in collaboration with regional and local governments. The guideline, ‘Guide for Radon Measurements in Residential Dwellings (Homes),’ was published in 2008 and included improvement measures to reduce radon level [5]. It was approved by the Radiation Protection Committee of the federal government, provincial and territorial governments in December, 2006, and accepted by the Canadian government on June 9, 2007. The radon measurement method used in Canada is almost the same as the one used in the US, involving long-term and short-term measurements.

Radon level in homes or buildings can vary drastically with time. Within a day, radon level in a house can change drastically due to several factors including seasonal changes. The highest radon level is generally observed during winter. Investigating the results, the long-term measurement method was evaluated to ensure a more appropriate method for measuring annual average of radon level than the short-term method. Health Canada recommends long-term (3–12 months) measurements for radon testing at homes [6].

In rare cases, radon level needs to be determined quickly. In such cases, short-term measurements with a period of less than 3 months (generally 2–7 days) are taken. Testing periods of less than 2 days should not be approved for the purpose of evaluating necessity of improvement plans. As mentioned above, radon levels change with time, and hence short-term measurement results should be confirmed with secondary long-term measurements. Secondary testing must be performed at the same location as that of primary testing. Since short-term measurements over a period of a few days do not provide sufficient results for radon reduction, secondary testing is required. Secondary testing is always required to reach a decision on whether radon reduction should be performed regardless of primary test results.

Short-term measurements must be taken in sealed buildings to stabilize radon level and increase the validity of estimations of annual average of radon level. The building must remain sealed throughout the measurement period, and for a period of over 4 days, the building must be sealed for 12 h before the start of the measurement.

Short-term measurements should not be taken during severe storms or abnormally high winds. Rapid changes in atmospheric pressure due to storms can alter the speed of radon inflow and increase the possibility of drastic differences in indoor and outdoor atmospheric pressure. Fast winds and differences in atmospheric pressure increase the possibility of changes in radon levels. Sealed buildings are generally measured initially in cold seasons, between October and April.

### The United Kingdom

In the UK, long-term measurements using a passive radon detector is the most appropriate technology for validating radon levels in residences that are higher than the recommended value by the HPA and the government [7]. As short-term measurement is generally not accepted as appropriate evidence for evaluating radon safety, it is not included in the radon evaluation system of the United Kingdom.

To measure the annual average of radon level for the purpose of comparing with the appropriate radon level, a proper measurement method must be applied as per the official test process. All measurements should be made through a detector in the bedroom or main living space of a residence over a period of at least 3 months. To test the effectiveness of reduction, measurements must be taken at the same location where previous measurements were taken before installing a reduction facility. Detectors must be designed to exclude radon daughters. In the UK, residents can apply online or by phone for radon detectors and receive them via mail for personal detection. Therefore, guidelines of a comprehensive overview of measurements must be provided to the applicants.

A detector can be placed on a shelf or furniture in a living room. If possible, it should be placed near an inner wall, avoiding hallways, areas exposed to direct sunlight, areas with drafts, areas close to a heat source, or inside another object (especially furniture). Further, window frames, radiators, fireplaces, televisions, and electronics generating heat should be avoided. Although the detector is not dangerous, it should be out of reach of children or animals.

Similar considerations should be taken when the detector is installed in a bedroom. If constructions are planned or instructions have not been followed, one must contact the detector provider immediately and follow the recommendations rather than try to store the detector.

After measuring for approximately 3 months, the detector is sent back to the provider along with a sheet containing data from the start date to the end date for analysis. After a few months, the residents receive results of the measurements take at their places of residence.

## Conclusion

In this study, the official test process of Korea was compared to that of other countries. Both short-term and long-term measurement methods are used in all countries investigated in this study including Korea, while the UK recommends only long-term measurement. Further, while information on measurement devices is provided on most official test processes, that of the UK does not contain information on recommended devices. However, websites concerning radon in the UK provide information on such devices.

The recommended season for measurements is specified only in the official test process of Canada (October to April). The recommended radon level in the US is 148 Bq/m^3^, which is the same as in Korea, while that in Canada and the UK is 200 Bq/m^3^. In particular, the UK separates recommendation standards between new and existing buildings, in which the 200 Bq/m^3^ limit is only a recommendation for existing buildings, while it is a requirement for new buildings.

While measurement location is specified in the official test processes of the US and Canada, that of the UK contains only general information on location selection without specifics. The official test process of Korea does not specify measurement location. Further, seasonal adjustment or temperature adjustment values are only used in the UK.

This study investigated and compared official radon test processes in the Republic of Korea with those of three other countries. The radon measurement method used in Korea comprises only one step. This study can be used as supporting data for standardization of radon measurement protocols in Korea and provision of improvement plans. A comparison of the official radon test processes in Korea with those of three other countries is summarized in Table 1.

**Table 1:** Comparison between Korea and other countries

Country	Measurement Method	Measurement Season	Recommended Level (Bq/m^3^)	Measurement location (cm)						Adjustment values
				Floor	Ceiling	Height	Other objects	Door or window	Outer wall	
The US	Short-term/Long-term	-	148	50	-	200–250	10	90	30	-
Canada	Short-term/Long-term	October to April	200	-	50	80–200	20	-	50	-
The UK	Long-term	-	200	-	-	-	-	-	-	○
Korea	Short-term/Long-term	-	148	-	-	-	-	-	-	-
